# Electrospun MXene/polyimide nanofiber composite separator for enhancing thermal stability and ion transport of lithium-ion batteries

**DOI:** 10.3389/fchem.2025.1555323

**Published:** 2025-02-21

**Authors:** Yitian Wu, Wenhui Wei, Tianxue Feng, Wenwen Li, Xiaoyu Wang, Tao Wu, Xingshuang Zhang

**Affiliations:** Advanced Materials Institute, Qilu University of Technology (Shandong Academy of Sciences), Jinan, China

**Keywords:** lithium-ion batteries, separator, thermal stability, polyimide, nanofibers, MXene

## Abstract

Safety of lithium-ion batteries (LIBs) has garnered significant attention. As an essential component of batteries, the separator plays a crucial role in separating the positive and negative electrodes, preventing short circuits, and allowing ion transport. Therefore, it is necessary to develop a high-performance separator that is both thermally stable and capable of rapid Li^+^ transport. Polyimide (PI) is a material with high thermal stability, but low electrolyte wettability and high interfacial resistance of PI restrict its application in high-performance LIBs batteries. MXene possesses excellent mechanical properties and good electrolyte affinity. PI/MXene nanofiber composite separator. Combines the high thermal stability of PI with the superior electrolyte wettability of MXene. It exhibits a high tensile strength of 19.6 MPa, low bulk resistance (2.5 Ω), and low interfacial resistance (174 Ω), as well as a low electrolyte contact angle of 29°, while retaining the high-temperature resistance and flame retardancy of PI. Batteries assembled with this composite separator demonstrated a specific capacity of 111.0 mAh g^−1^ and a capacity retention rate of 66% at 2C. In long-term cycling tests of LiFePO₄ half-cells at 1C, after 200 charge-discharge cycles, the PI/MXene battery showed a discharge specific capacity of 126.7 mAh g^−1^ and a capacity retention rate of 91%. Additionally, the battery operated normally at 120°C. The composite separator, by integrating the high thermal stability of PI with the excellent electrolyte wettability and conductivity of MXene, demonstrates significant advantages in enhancing battery safety and cycling performance. Through this composite structure can provide a more reliable and safe solution for high-performance LIBs.

## 1 Introduction

In recent years, lithium-ion batteries (LIBs) have been widely used in fields such as smartphones, new energy vehicles, digital cameras, and grid energy storage systems due to their low self-discharge, lack of memory effect, and long lifespan (Arifeen et al., 2021; Ahn et al., 2019; Babu et al., 2018). The separator, as an essential component of the battery, serves to separate the anode from the cathode, preventing their direct contact while providing a fast and stable pathway for the movement of ionic charges in the electrolyte (Bai et al., 2018; Zhai et al., 2021). However, due to the relatively high cost of raw materials and the complexity of manufacturing processes, the cost of the separator exceeds 20% of the total cost (Guo et al., 2019).

The main component of lithium-ion battery separators is typically polyolefins. Polypropylene (PP) and polyethylene (PE) have become the primary commercial battery separators due to their high cycling stability. However, their inherent drawbacks, such as poor wettability, low liquid absorption, and inadequate thermal stability, affecting the performance and safety of the battery (Thangarasu et al., 2021; Yan et al., 2022). Micro-porous polyolefin separators and multilayer composite separators are widely used for their chemical stability and thermal shutdown functionality, such as Celgard 2400. While, they still face challenges under high-temperature operating conditions, including insufficient thermal stability, limited electrolyte wettability and ionic conductivity, and weak mechanical strength, which can easily lead to internal short circuits, which can compromise battery safety (Almuhamed et al., 2016). Furthermore, the low melting point of PP and PE separators makes them susceptible to melting and shrinking at high temperatures, increasing the risk of rapid heat accumulation and explosion. Therefore, there is an urgent need to develop advanced battery separators with excellent thermal stability, high porosity, and high electrolyte absorption capacity, as well as the ability to rapidly transport ions, to meet the growing demands of high-performance LIBs (Song et al., 2023; Parsaei et al., 2022; Yang et al., 2022).

PI is a high-performance polymer material known for its excellent thermal stability, chemical resistance, and mechanical strength. Composed of imide groups, PI possesses a tunable molecular structure and a unique polymer crystalline structure (Wei et al., 2023). It is widely used in aerospace, electronics, optics, and electrical fields. In lithium-ion batteries, PI is also employed as separator material. Compared to traditional organic and inorganic separators, PI offers high heat resistance and outstanding chemical stability. Additionally, PI separators fabricated using electrospinning technology exhibit high porosity and favorable ion permeability (Guo et al., 2023; Ilyas et al., 2024; Wang et al., 2023; Yan et al., 2016; Xingshuang et al., 2021).

In this work, the PAA solution was synthesized using two diamines, 2, 4, 6-trimethtivity, superior mechanical properties, and chemical stability, aims to leverage the functional groups of MXene to enhance electrolyte affinity and ionic conductivity, as well as to utilize its unique two-dimensional structure to improve the mechanical properties of the separator (Gogotsi, 2023; Gogotsi and Anasori, 2019; Lei et al., 2015). The two-dimensional framework structure of MXene effectively improves the mechanical properties of the separator (Lee and Kim, 2021), thereby preventing potential accidents during battery operation, such as lithium dendrite penetration. This is crucial for ensuring the safety and reliability of the battery. Additionally, the incorporation of MXene within the fibers enhances ionic conductivity, reduces interfacial and bulk resistance, thereby improving overall efficiency and performance (Hyokyeong et al., 2024; Li et al., 2023; Ming et al., 2020; Wang et al., 2024). The combination of these properties makes the PI/MXene composite separator a promising candidate for high-performance lithium-ion batteries, addressing the limitations of traditional separators and providing a more robust and efficient solution.

## 2 Experiment

### 2.1 Materials

TMPDA (98%), DDM (99%), LiCl (99%), PMDA (98%, dried at 120°C for 6 h), N, N-Dimethylacetamide (DMAC, ≥99.8%), HF (40%) were purchased from Shanghai Macklin Biochemical Co., Ltd., China. Al-Ti_3_C_2_AlC_2_ (300 mesh, ≥99.9%) was purchased from FoShan XinXi Technology Co., Ltd., China. HCl (38%) was purchased from Laiyang Economic and Technological Development Zone fine chemical plant.

### 2.2 Preparation of PI/MXene nanofiber membrane

#### 2.2.1 Synthesis multilayer antioxidant MXene

2 g Al-Ti_3_C_2_AlC_2_ was added 8 times to a mixed solution of 43.2 mL 10M HCl, 10.8 mL deionized water, and 6 mL 40% HF, and stirred at 35°C for 24 h. The resulting mixture was centrifuged and washed until the pH > 6 of the supernatant. Then, 1 g LiCl was dissolved in 50 mL deionized water and added to the precipitate from the previous step. The mixture was stirred at room temperature for 8 h, followed by centrifugation, filtration, and vacuum drying, yielding multilayer antioxidant MXene (Chen et al., 2021; Wang et al., 2022; Yali et al., 2021; Zhou et al., 2024).

#### 2.2.2 Preparation of PI/MXene separations

Two binary amine monomers, 0.2212 g TMPDA and 0.5769 g DDM, were added to 5 mL DMF solution and stirred for 2 h. 0.0875 g MXene was then added to the solution and ultrasonically dispersed for 2 h. Subsequently, 0.9519 g PMDA was added in 8∼10 aliquots. The mixture was stirred at 5°C under a nitrogen atmosphere for 10∼12 h, resulting in PAA/MXene solution. Notably, the preparation of the PAA solution follows the aforementioned description but omits the step of adding MXene. The molar ratio of the two binary amine monomers to the binary anhydride monomer is 1:1.02, and MXene constitutes 1∼5% of the total mass of the two binary amine monomers and the binary anhydride monomer.

The PI (or PI/MXene) nanofibrous membrane is fabricated via a two-step process encompassing electrospinning and thermal imidization (Cao et al., 2016; Li et al., 2019a). The viscous PAA (or PAA/MXene) solution is loaded into a 10 mL disposable syringe equipped with an 18 G metal needle for electrospinning. The parameters for the electrospinning, including the injection rate, relative humidity, applied voltage, and the distance between the needle tip and the collecting drum, are set to 0.0010 mm/s, 60∼70%, 25 kV, and 20 cm, respectively. Subsequently, the as-prepared PAA (or PAA/MXene) nanofibrous membrane is dried at 60°C for 8 h. The thermal imidization process in an air atmosphere follows: the temperature is initially increased to 180°C at a rate of 10°C/min and maintained for 1 h to eliminate residual solvents; it is then further elevated to 250°C at the same heating rate and held for 1 h. Finally, the temperature is raised to 350°C at a rate of 5°C/min and sustained for 1 h. Ultimately, the resulting product is a PI (PI/MXene) nanofibrous membrane.

### 2.3 Characterization of the physical properties of PI/MXene separators

The chemical structure and functional groups of PI and PI/MXene was determined by Fourier transform infrared (FTIR, VERTEX 70, Germany). The morphology of the synthesized nanofibers membrane was characterized by SEM (JEOL, 15 kV) and High-Resolution Transmission Electron Microscopy (HRTEM, FEI Talos F200s, United States). The thermal stability of the prepared separators, was conducted by thermogravimetric analyzer (TGA/DSC3+, METTLER TOLEDO), analysis was at temperature from 25°C to 900°C under air atmosphere. The crystal structure of samples was analyzed by X-ray diffraction technique (XRD, smartlab, Japan) in the 2θ ranges of 10-90^o^. Mechanical properties of the prepared separators were measured by tensile and compression analyzer (UTM2102, Shenzhen, China). Whereas, the separator’s ability to absorb electrolytes was measured using contact angle machine (SDC-100, SINDIN, China). The porosity of the separator is determined through the uptake of n-butanol and calculated according to the following Equation 1 (Tan et al., 2018).

$$P\text{orosity}\%=\frac{W_{b}‐W_{a}}{\rho_{d}\times V}\times 100\%$$ (1)

Where w_a_ represents the absolute mass of the dry membrane, w_b_ is the mass of the membrane after absorption of n-butanol, and ρ_d_ is the density of n-butanol. V is the apparent volume of the membrane. The absorption rate is measured by immersing the separator in an electrolyte for 2 h and calculated using the Formula 2 (Cai et al., 2020).

$$\text{Electrolyte uptake}\%=\frac{W_{1}‐W_{2}}{W_{2}}\times 100\%$$ (2)

Where w_2_ represents the weight of the fully dried separator, and w_1_ represents the weight of the immersed separator recorded every 10 min. The electrolyte retention rate of the separator is calculated using the following Equation 3 (Wang et al., 2017).

$$\text{Electrolyte retention}\%=\frac{W_{x}‐W_{2}}{W_{1}‐W_{2}}\times 100\%$$ (3)

Where w_x_ denotes the mass of the separator fully immersed in the electrolyte, recorded every 10 min within the first 100 min and then every 50 min thereafter.

### 2.4 Electrochemical measurements

An electrochemical workstation (CHI 760E, Chenhua Instruments Co., Ltd., Shanghai, China) was used for the measurements of ionic conductivity. The samples were fully immersed in the liquid electrolyte and sandwiched between two stainless steel washers (SS), then assembled into a symmetric coin cell with the configuration “SS/separator/SS.” The frequency range for the tests was set from 0.1 to 10^4^ Hz, with an amplitude of 5 mV. The ionic conductivity (σ) was calculated as follows Formula 4 (Li et al., 2019b):

$$\sigma =\frac{d}{R_{b}\times S}\times 100\%$$ (4)

where d is the thickness of the separator, R_b_ is the bulk resistance of the separator and electrolyte system, and S is the contact area of the separator.

To test the interfacial resistance between the separator and Li metal, the separator soaked in the electrolyte was sandwiched between two Li metal electrodes and assembled into a “Li/separator/Li” symmetric coin cell. The frequency range for the tests was set from 0.02 to 10^6^ Hz.

**FIGURE 1:**
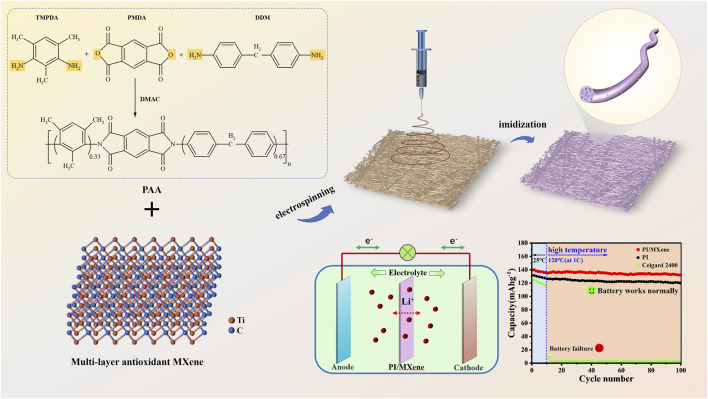
Preparation, process diagram of PI/MXene nanofiber membrane and application in LIBs separator.

Using a battery testing system (CT-4008, Shenzhen New Energy Co., Ltd., China), the voltage ranges were set to 2.5∼4.2 V and 3.0∼4.45 V. The cycling performance of the “LiFePO_4_/separator/Li” half-cell was evaluated by charging and discharging for 200 cycles at 1 C (1 C = 170 mAh g^−1^). The rate performance of the half-cell was assessed by charging and discharging continuously for 5 cycles at various rates (charged at 0.2C and discharged at 0.1, 0.2, 0.5, 1, 1.5, and 2 C, then returning to 0.1 C). To evaluate the battery performance under high-temperature conditions, a “LiFePO_4_/separator/Li” half-cell was assembled. The cell was cycled for the first ten cycles at 25°C, followed by cycling up to 100 cycles at 120°C (Figure 1).

## 3 Results and discussion

### 3.1 Analysis and characterization of PI/MXene membrane

**FIGURE 2:**
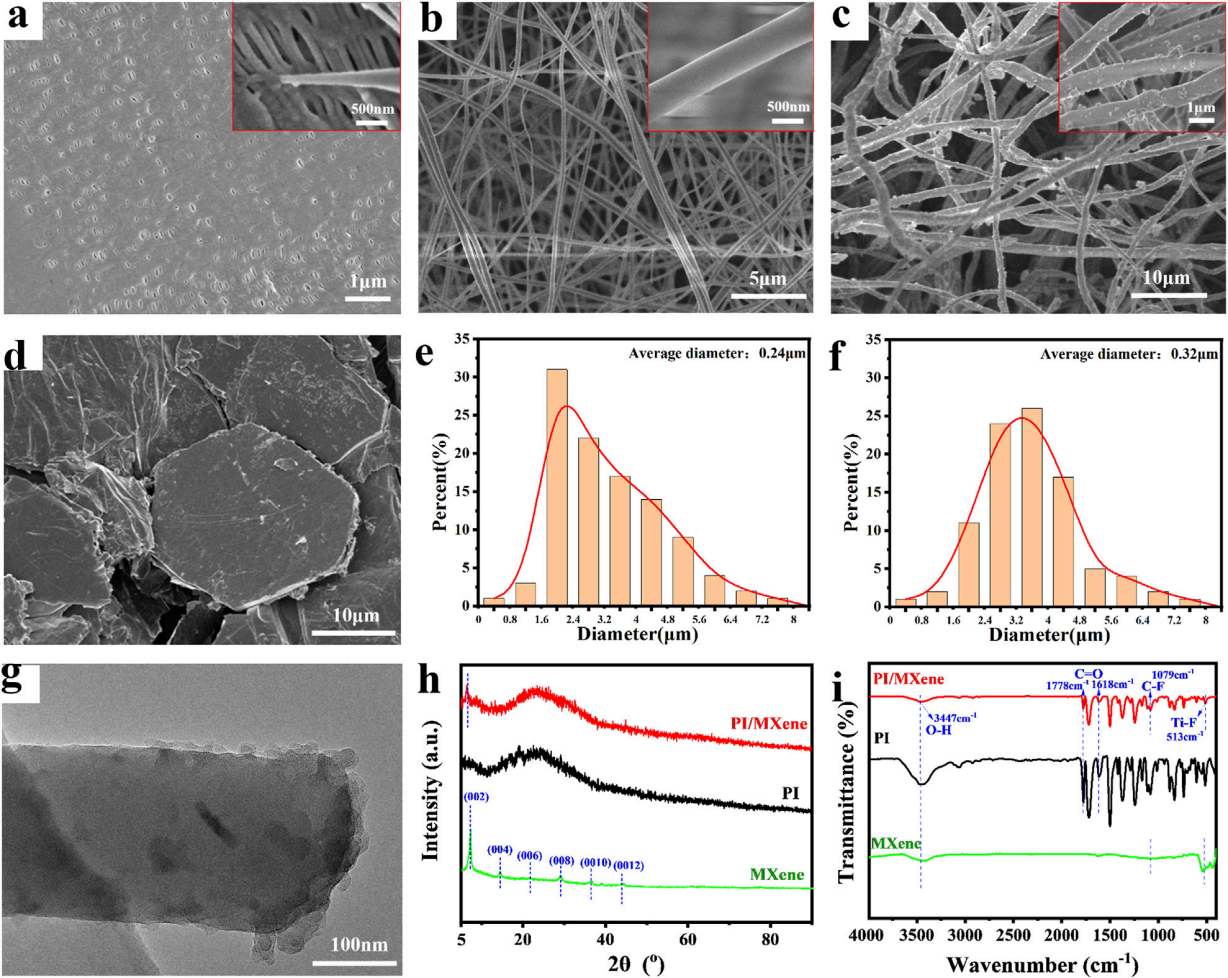
**(A)** SEM images of Celgard 2400, **(B)** PI, **(C)** PI/MXene and **(D)** MXene, **(E)** Diameter distributions of the PI and **(F)** PI/MXene. **(G)** TEM images of PI/MXene. **(H)** XRD pattern and **(I)** FTIR spectrum of PI, MXene and PI/MXene.

Compared to the commercial separator, the fiber morphology of PI and PI/MXene separators exhibits significant differences. As shown in Figure 2A, the SEM image of the commercial separator Celgard 2400 reveals that its surface is covered with a dense film, which may result in limited porosity and consequently affect the permeation rate and conduction speed of charged ions in the electrolyte. The high-magnification SEM image also indicates that the fiber diameter of Celgard 2400 is relatively fine, approximately 50 nm. From Figures 2B, C, it can be observed that the fiber surface of PI separator is smooth and exhibits a certain degree of orientation due to the electrospinning process. This orientation enhances the mechanical properties in the corresponding direction. From the SEM image of MXene (Figure 2D), it can be observed that the prepared MXene exhibits a hexagonal multilayered flake structure. Therefore, the average fiber diameter of PI/MXene is 320 nm, which is larger than the average fiber diameter of PI (240 nm) (Figures 2E, F).

In contrast, the PI/MXene separator show protrusions on their fiber surface, which are attributed to the addition of MXene. As shown in the TEM image in Figure 2G, incorporating MXene during the synthesis of the PAA precursor modifies the structure of the PAA. After electrospinning and imidization, the MXene is fully doped within the fiber, and its angular structure results in the observed protrusions in the SEM images. This also likely contributes to the thicker appearance of the PI/MXene fibers compared to the PI fibers. This structural modification likely contributes to the larger fiber diameter of PI/MXene compared to that of pure PI (Figures 2E, F). Larger fiber diameters can provide better mechanical strength and thermal stability, but they may also affect the porosity and electrolyte absorption capacity of the separator. Therefore, optimizing the content and distribution of MXene is crucial for balancing these properties.

During the electrospinning process, various spinning parameters such as PAA concentration, humidity, and collector rotation speed significantly influence the formation of PI (PI/MXene) nanofibers, as shown in Supplementary Figure S1. Firstly, if the PAA concentration is too low, the spinning solution becomes either too dilute or too viscous, making it impossible to produce fibers under the applied voltage. Additionally, when the humidity is below 60% or above 80%, the resulting PI membrane loses its fibrous structure and cannot form nanofibers. According to the experimental results, the optimal PAA concentration for electrospinning is 35%. Other optimal spinning parameters are detailed in Section 2.2.2.

According to Table 1, the commercial Celgard 2400 separator has the lowest thickness of 32 μm, while the PI and PI/MXene separators have thicknesses of 87 μm and 93 μm, respectively. Although the thickness of the separator can be controlled through electrospinning, an excessively thick separator may lead to delamination, resulting in non-uniform and incomplete imidization. Also, a thicker separator can reduce the ion migration rate. On the contrary, an overly thin separator may compromise mechanical properties, making it more susceptible to puncture by lithium dendrites, which can cause battery short-circuiting. Finally, the PI separator and PI/MXene separator prepared by electrospinning exhibit higher porosity compared to the commercial Celgard 2400 separator (Table 1), which also results in a lower density compared to the commercial Celgard 2400 separator (Yuan et al., 2021; Zhu et al., 2020).

**TABLE 1:** Physicochemical properties of Celgard 2400, PI and PI/MXene separators.

Types	Thickness (μm)	Density (g·cm^−3^)	Porosity (%)	Electrolyte uptake (%)	Electrolyte retention (%)	Contact angle (°)	Ionic conductivity (mS·cm^−1^)
Celgard 2400	32	0.38	42	203	35	39	0.17
PI	87	0.21	93	725	82	23	0.45
PI/MXene	93	0.24	76	832	85	7	0.53

As shown in Figure 2H, the XRD pattern of MXene displays six prominent diffraction peaks, confirming its hexagonal structure, with the (002) peak being the most pronounced. By comparing the XRD diffraction patterns of the PI separator before and after MXene doping, it is evident that the PI/MXene composite shows a diffraction peak at 2θ ≈ 6.8°, which corresponds to the (002) peak of MXene (Karlsson et al., 2015; Mostafaei and Heidari Semiromi, 2022). This peak is absent in the XRD pattern of the pure PI separator, confirming the successful incorporation of MXene into the PI nanofibers. Furthermore, FTIR spectra in Figure 2I indicate that the infrared spectrum of PI/MXene shows a broad peak at 3,447 cm^−1^, representing O-H stretching vibrations (from both PI and MXene). Strong peaks are observed at 1,778 cm^−1^ and 1,619 cm^−1^, corresponding to C=O stretching vibrations (from PI). Additionally, peaks at 1,079 cm^−1^ and 513 cm^−1^ are attributed to C-F and Ti-F stretching vibrations (from MXene), respectively. It can be observed that the characteristic peaks of PI still exist in the doped sample, but with some variations in intensity. The characteristic peaks of MXene also appear in the doped sample. Combined with the analysis of the XRD patterns, these results confirm the successful incorporation of MXene into the PI nanofibers (Li et al., 2025; Zhang et al., 2023; Liang et al., 2016; Liu et al., 2020). Furthermore, according to the EDS elemental mapping image of PI/MXene (Supplementary Figure S2), it can be found that the distribution of Ti and Al elements exists inside the PI fibers. These elements are not present in PI but are in MXene, thus confirming the successful doping of MXene.

### 3.2 Performance analysis of PI/MXene separators

#### 3.2.1 Mechanical performance of PI/MXene separators

**FIGURE 3:**
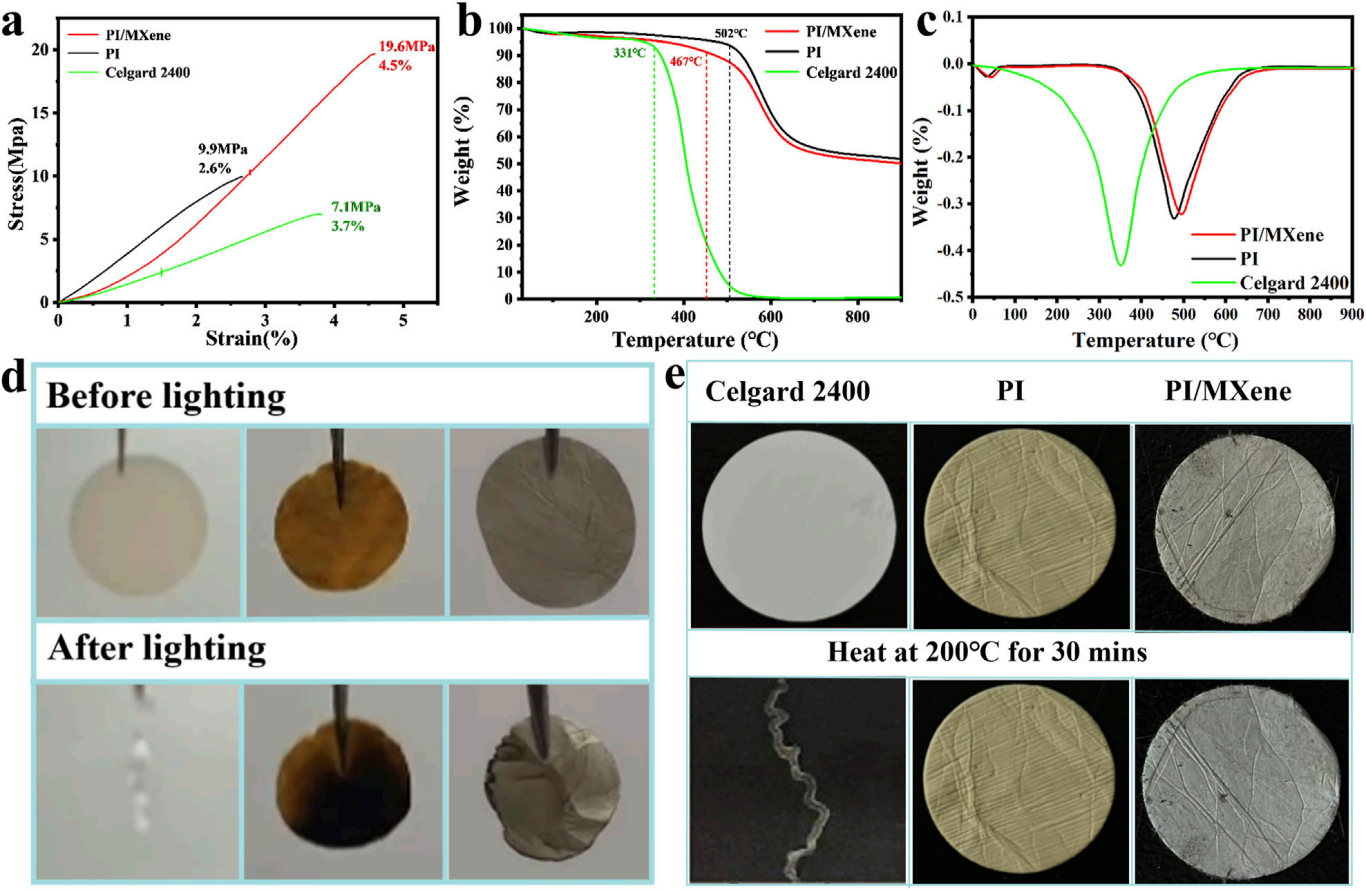
**(A)** Stress-strain curves of Celgard 2400, PI, and PI/MXene separators; **(B)** TGA curves of Celgard 2400, PI, and PI/MXene separators; **(C)** Derivative Thermogravimetry (DTG) curves of Celgard 2400, PI, and PI/MXene separators; **(D)** ignition tests of Celgard 2400, PI, and PI/MXene separators; **(E)** thermal stability tests of Celgard 2400, PI, and PI/MXene separators.

Figure 3A shows the mechanical properties of PI, PI/MXene, and Celgard 2400 separators. The comparison clearly indicates that the commercial Celgard 2400 separator exhibits the poorest mechanical performance (7.1 MPa, 3.7%), while the PI separator demonstrates better performance (9.9 MPa, 2.6%). The PI/MXene separator displays the best mechanical performance (19.6 MPa, 4.5%).This can be attributed to the inherently good mechanical properties of PI, which are superior to those of the Celgard 2400 separator. Additionally, the layered structure of MXene also possesses excellent mechanical properties. The incorporation of MXene into the PI matrix slightly enhances its mechanical performance.

The mechanical properties of PI/MXene at different temperatures and with varying doping amounts are shown in Supplementary Figure S3. As the temperature increases, the deepening of imidization of PI, leading to a more compact intermolecular structure and an improvement in its mechanical properties. With increasing MXene doping, the mechanical properties of PI first increase and then decrease, reaching a peak at 5% doping. This is because an appropriate amount of MXene, when incorporated into the PI separator, can enhance the composite’s strength due to the high mechanical strength of MXene itself. However, excessive MXene can disrupt the microstructure of PI or block the pores within the PI nanofibers, leading to a decline in mechanical properties. Notably, we measured the stress-strain curves of PAA, pure PI, and PI/MXene composite separators containing 0%, 1%, 5%, 9%, and 12% MXene (Supplementary Figure S4). The results show that PAA, which has not undergone imidization, exhibits higher mechanical strength. In contrast, the mechanical properties of PI and PI/MXene composites decrease after thermal imidization. As the MXene content increases, the mechanical performance of the composite separator initially improves and then decreases, reaching its maximum value at 5% MXene content. However, when the MXene content exceeds a certain threshold, the excess MXene leads to a reduction in the porosity of the separator, which in turn affects its mechanical performance. Specifically, an excessive amount of MXene fills the pores in the separator, reducing the porosity and consequently decreasing the mechanical performance of the separator. Therefore, the optimal MXene doping amount was determined to be 5%.

#### 3.2.2 Thermodynamic performance analysis of separators

According to the TGA and DTG analysis shown in Figures 3B, C, the commercial Celgard 2400 separator begins to decompose at around 331°C and is completely decomposed by approximately 589°C. In contrast, the PI separator and the PI/MXene separator begin to decompose at around 502°C and 467°C, respectively. At 900°C, they still retain 50.1% and 48.9% of their original mass, respectively. This high thermal stability can be attributed to the highly stable aromatic ring structure of PI, which resists thermal decomposition at high temperatures. The presence of aromatic rings makes the molecular chains more rigid and stable, thereby enhancing the thermal stability of the material. The addition of MXene does not alter this high-temperature resistance. Furthermore, the Celgard 2400 separator shows a distinct endothermic peak at around 331°C (Figure 3C). In contrast, no significant endothermic peaks are observed for the PI and PI/MXene separators in the temperature range of 100°C–350°C. The endothermic peak at around 45°C is attributed to the evaporation of moisture in the separators.

During the ignition and high-temperature resistance tests (Figures 3D, E), the Celgard 2400 separator immediately exhibited a severe combustion reaction upon contact with the heat source, completely losing its original appearance. It also gradually shrank during the heating test and ultimately lost its original shape. In contrast, the PI/MXene separator maintained similar thermal resistance and flame retardancy to the PI separator. No smoke was observed during the ignition and heating processes. However, there is a slight shrinkage phenomenon. Therefore, the flame retardancy of the PI/MXene separator is slightly worse than that of the PI separator.

This high-temperature resistance is of significant importance in the operation of lithium-ion batteries. The thermal stability enhances the battery’s ability to withstand heat generation. Even in the event of overheating, the flame-retardant properties of the PI/MXene composite separator prevent incidents such as fires and explosions, thereby ensuring the safety of the battery.

#### 3.2.3 Analysis of liquid absorption rate and contact angle of separators electrolyte

In addition to affecting ion transport efficiency, the excellent electrolyte absorption and retention rates, can also significantly enhance the overall performance of the battery. Specifically, high electrolyte absorption ensures that the separator rapidly and fully absorbs the electrolyte, thereby reducing the internal resistance of the battery and improving its charge and discharge rates. Furthermore, good electrolyte retention prevents the gradual loss of electrolyte during battery cycling, thus maintaining the stability and extending the cycle life of the battery. These properties collectively contribute to not only increasing the energy density and power density of the battery but also enhancing its reliability and stability under various operating conditions. Therefore, optimizing electrolyte absorption and retention rates is a key factor in improving the performance of lithium-ion batteries.

**FIGURE 4:**
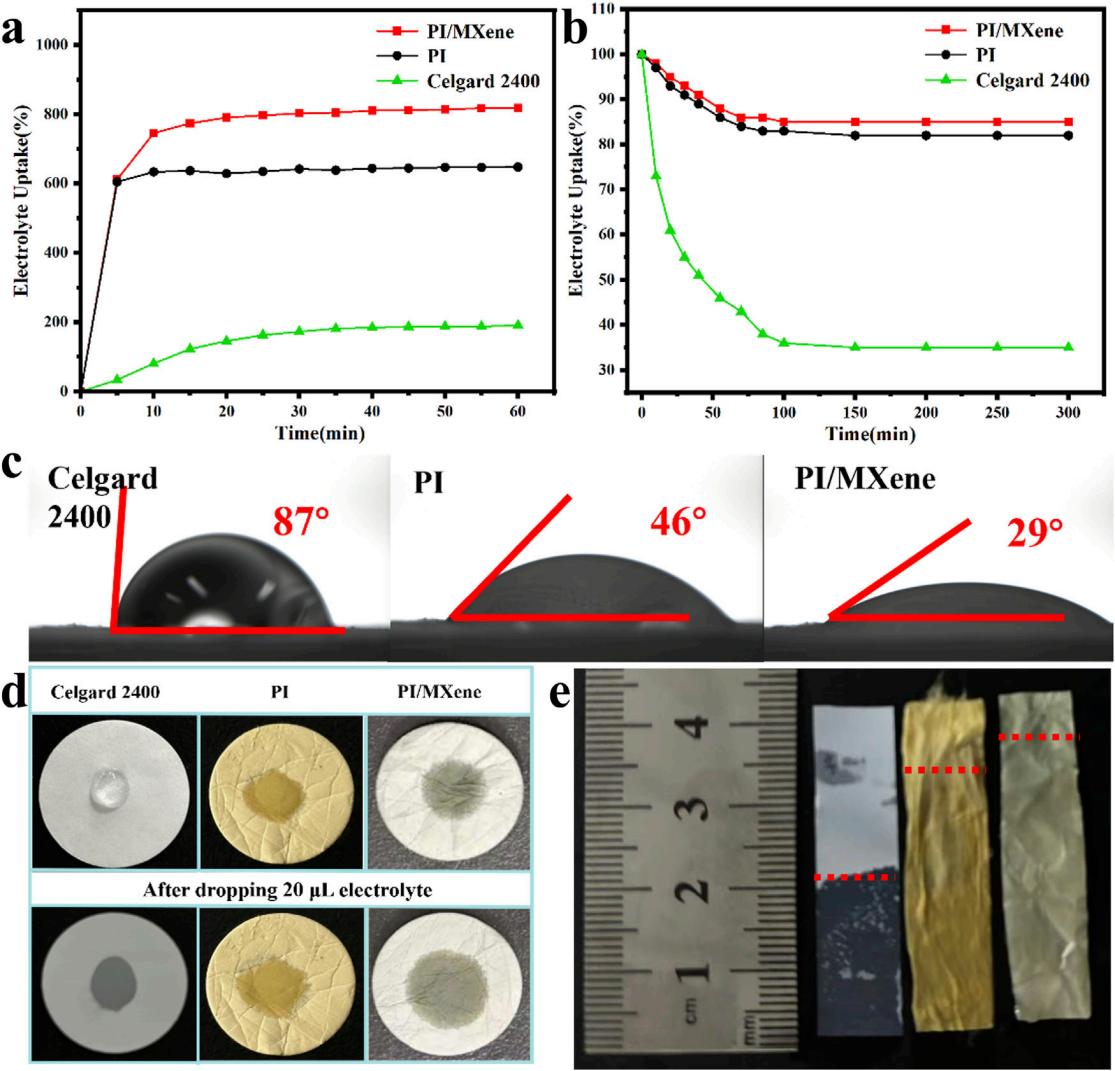
**(A)** Electrolyte absorption and **(B)** electrolyte retention of Celgard 2400, PI, and PI/MXene separators; **(C)** contact angle of Celgard 2400, PI, and PI/MXene separators with liquid electrolyte; **(D)** electrolyte droplet drop test on Celgard 2400, PI, and PI/MXene separators; **(E)** electrolyte immersion height on Celgard 2400, PI, and PI/MXene separators.

The electrolyte absorption rate and retention rate of the three separators were tested. According to Figures 4A, B; Table 1, the absorption rate increases over time for three separators. The absorption rates of the Celgard 2400, PI, and PI/MXene separators stabilize at 203%, 725%, and 832%, respectively. The retention rate, on the other hand, decreases over time and stabilizes at 35%, 82%, and 85% for the Celgard 2400, PI, and PI/MXene separators, respectively. The contact angles of the electrolyte on Celgard 2400, PI and PI/MXene separators are 79°, 46°, and 29°, respectively (Figure 4C). Further, the contact angles of different MXene doping amounts were tested (Supplementary Figure S5). As the MXene doping amount increases, the contact angle decreases. This is attributed to the presence of electrolyte-philic functional groups in MXene, which enhance the electrolyte affinity of the PI/MXene composite. However, considering the mechanical and thermal properties of the separator, it is not advisable to use a higher content of PI/MXene indiscriminately. Overall, 5% MXene content appears to be the most suitable.

The images of electrolyte diffusion on the three types of separators are shown in Figure 4D. The Celgard 2400 separator shows no significant change in electrolyte diffusion from 0 to 5 s, whereas both the PI and PI/MXene separators exhibit greater diffusion at 0s compared to the Celgard 2400 separator, with further diffusion observed at 5 s. The PI/MXene separator demonstrates superior electrolyte absorption compared to the PI separator. This is further confirmed by the electrolyte absorption height in Figure 4E, where the Celgard 2400 separator has an absorption height of 22 mm, while the PI and PI/MXene separators have absorption heights of 34 mm and 37 mm, respectively.

Overall, the excellent electrolyte affinity of the PI/MXene separator enables faster wetting by the electrolyte, thereby reducing the initial activation time after battery assembly. The good electrolyte affinity and high ionic conductivity of the PI/MXene separator facilitate faster Li^+^ ion transport through the separator, lowering the internal resistance of the battery and enhancing the charge-discharge rate and power density. The superior electrolyte uptake and retention capabilities ensure uniform distribution of the electrolyte within the separator, preventing localized dry areas and improving the consistency and stability of the battery. This, in turn, enhances the energy density of the battery.

### 3.3 Electrochemical performance of PI/MXene separators

**FIGURE 5:**
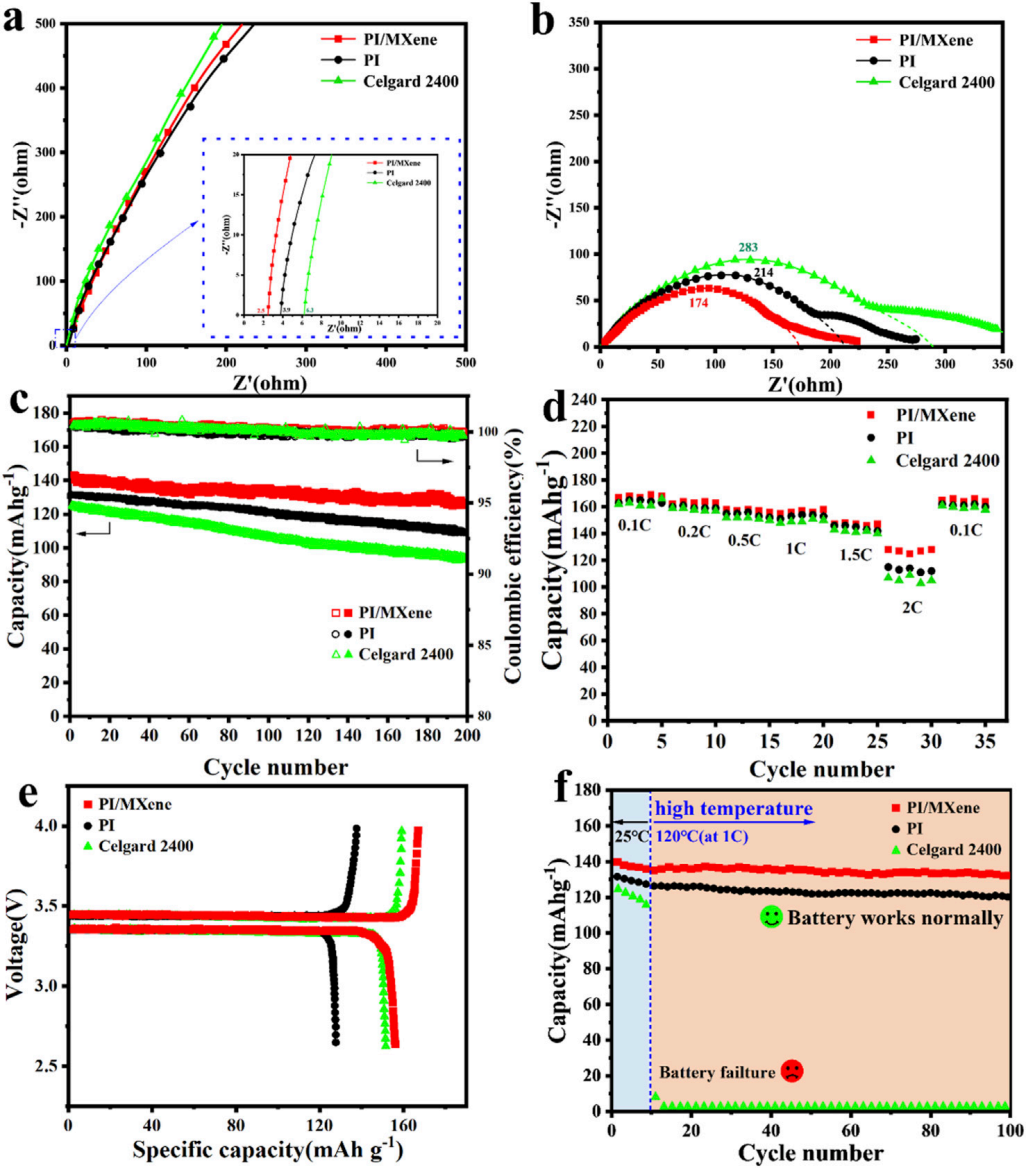
The Nyquist plots of **(A)** “SS/Separator/SS” cells and **(B)** “Li/Separator/Li” cells using Celgard 2400, PI and PI/MXene separators; **(C)** Cyclic tests and Coulombic efficiency at 1C and **(D)** rate performance tests for the “LiFePO_4_/Separator/Li” cells using Celgard 2400, PI and PI/MXene separators; **(E)**Initial charge-discharge capacities Li–LiFePO_4_ cells with liquid-electrolyte -socked separator; **(F)** cyclic tests at 1 C under 120°C for the “LiFePO_4_/Separator/Li” cells using Celgard 2400, PI and PI/MXene separators.

To test the electrochemical performance of the PI/MXene separator, full cells, half cells, etc., were assembled. The data in aspects such as interfacial impedance, rate performance, and Coulombic efficiency were tested (Figure 5), and our battery did not experience a short circuit during operation.

The Nyquist plots of the “SS/separator/SS” cells from EIS tests (Figure 5A) show that the PI/MXene composite separator cell has the lowest bulk resistance of 2.5 Ω. In contrast, the bulk resistances of the PI and Celgard 2400 cells are 3.9 Ω and 6.3 Ω, respectively. The ionic conductivity calculated using Equation 4 for the PI/MXene composite separator is 0.53 mS cm⁻^1^ (Table 1), which is higher than that of the commercial Celgard 2400 separator (0.18 mS cm⁻^1^). The Nyquist plots of the “Li/separator/Li” cells (Figure 5B) show that the PI/MXene composite separator cell also has the lowest interfacial resistance (174 Ω). Significantly lower than that of the PI separator (214 Ω) and the Celgard 2400 separator (284 Ω). The significant reduction in both bulk resistance and interfacial impedance of the PI/MXene composite separator can be attributed to the following reasons: firstly, the presence of functional groups such as -OH on the MXene surface and -COOH on the PI surface forms hydrogen bonds with the proton-accepting groups (ether and carbonyl groups) in the carbonate electrolyte, significantly enhancing the stability of the separator itself; sencondly the high polar compatibility between the PI/MXene composite separator fibers and the electrolyte components leads to increased van der Waals forces. This hydrogen bond structure and the enhanced van der Waals forces greatly improve the affinity and wettability between the separator and the electrolyte. Consequently, the enhanced electrolyte affinity and wettability of the PI/MXene separator provide optimized mass transfer channels for Li-ion migration, thereby reducing both the bulk resistance and interfacial resistance.

In the subsequent long-term cycling test of LiFePO₄ half-cells (Figure 5C), after 200 charge and discharge cycles at 1 C, the PI/MXene cell exhibited the highest discharge specific capacity of 126.7 mAh g⁻^1^ and a capacity retention rate of 91%. In stark contrast, the PI cell and Celgard 2400 cell showed discharge specific capacities and capacity retention rates of (113.1 mAh g⁻^1^, 86%) and (95.6 mAh g⁻^1^, 72%), respectively. The electrochemical performance of the PI/MXene composite separator was significantly improved due to the partial characteristics of MXene in the composite. MXene exhibits excellent electrolyte affinity, which enhances electrolyte wettability, reduces interfacial impedance, and increases ionic conductivity. It is hypothesized that the reduced charge transfer resistance and optimized lithium-ion transport pathways may contribute to better battery performance at higher charging and discharging rates.

As shown in Figure 5D, the PI/MXene composite separator battery exhibits a specific capacity of 107.0 mAh g^−1^ and a capacity retention rate of 63% at 2 C, which are significantly higher than those of the PI battery (90.4 mAh g^−1^ and 52%) and the Celgard 2400 battery (77.2 mAh g^−1^ and 48%). As illustrated in Figure 5E, the initial charge and discharge voltages were between 3.0 V and 4.3 V, conducted at a rate of 0.2 C. The initial discharge voltages for the Celgard 2400, PI, and PI/MXene separators were 3.956 V, 3.912 V, and 3.979 V, respectively, with a cutoff voltage of 2.7 V. The first discharge capacities for the Celgard 2400, PI, and PI/MXene separators were 152.7 mAh g^−1^, 139.3 mAh g^−1^, and 156.1 mAh g^−1^, respectively. This indicates that even at a low rate of 0.2 C, the PI/MXene separator can improve the discharge capacity. Since the PI/MXene separator absorbs more electrolyte than the Celgard 2400 separator, it exhibits better electrolyte storage and lithium ion transport performance. The excellent electrolyte wettability and interface stability contribute to the improved rate performance of the battery.

Furthermore, to further confirm the thermal stability of the separators in the batteries, cycling tests were conducted on LiFePO₄ half-cells assembled at 25°C for the first 10 cycles, followed by testing at 120°C. As can be seen in Figure 5F, the PI/MXene battery consistently provided higher capacities than the PI battery throughout all charge-discharge cycles, with no significant fluctuations observed after the abrupt temperature increase. In contrast, the Celgard 2400 battery nearly lost its capacity during the 11th cycle after the temperature increase due to the failure of the separator at such high temperatures, leading to an internal short circuit in the battery.

Compared to commercial Celgard 2400 and PI separators, the prepared PI/MXene composite separator exhibits excellent electrolyte wettability and affinity. This superior wettability and affinity significantly reduce the interfacial impedance between the separator and the electrolyte, thereby enhancing the ionic conductivity in the electrolyte. Consequently, LiFePO4 batteries assembled with the PI/MXene composite separator demonstrate better cycling and rate performance.Specifically, the high electrolyte wettability of the PI/MXene composite separator ensures that the electrolyte is uniformly and fully distributed throughout the separator, reducing the uneven distribution of the electrolyte within the battery and thus improving overall battery performance. Additionally, the low interfacial impedance means that ion transport between the separator and the electrolyte is more efficient, further enhancing the charge and discharge efficiency and cycle stability of the battery.

The PI/MXene composite separator offers significant advantages over previously prepared separators in several aspects. Firstly, its high-temperature resistance allows it to operate stably in high-temperature environments, enhancing the safety and reliability of the battery. Secondly, precise thickness control enables it to meet the requirements of different applications. Thirdly, the mechanical strength of the PI/MXene composite separator is significantly enhanced, which helps prevent damage to the battery due to mechanical stress during use. Finally, its excellent electrolyte adsorption capability ensures the effective utilization of the electrolyte, further improving battery performance. We compared some studies on the modification of PI separators (Supplementary Table S1), and found that the PI/MXene separator has the highest electrolyte absorption rate and porosity, while its thickness, tensile strength, and full-cell discharge capacity also have relatively excellent performances. The PI/MXene composite separator, with its superior high-temperature resistance, precise thickness control, enhanced mechanical strength, and excellent electrolyte adsorption, stands out as a highly promising candidate for high-performance lithium-ion battery separators.

## 4 Conclusion

By introducing MXene during the PAA synthesis process, followed by electrospinning and imidization, PI/MXene composite separator was successfully developed. The resulting PI/MXene separator not only retains the high-temperature resistance, flame retardancy, and high mechanical performance of the PI separator but also benefits from enhanced ionic conductivity and electrolyte affinity due to the presence of MXene. The tensile strength of the PI/MXene separator is 19.6 MPa. Additionally, PI/MXene separator exhibits better electrolyte wettability and affinity, with an electrolyte contact angle of 29°, and electrolyte uptake and retention rates of 832% and 85%, respectively. Consequently, LiFePO₄ lithium-ion batteries assembled with the PI/MXene composite separator demonstrate superior cycling and rate performance compared to those assembled with conventional PP and PI separators. For example, the LiFePO₄ half-cell based on the PI/MXene composite separator shows the highest initial discharge capacity (126.7 mAh g^−1^) and capacity retention (91%), significantly higher than those of the PI-based LiFePO₄ half-cell (113.1 mAh g^−1^, 86%) and the Celgard 2400-based LiFePO₄ half-cell (95.6 mAh g^−1^, 72%). This approach not only proves useful for the development of high-performance LIBs but also provides new insights and options for breakthroughs and innovations in a range of applications, including supercapacitors, lithium-sulfur batteries, and lithium metal batteries.

## Data Availability

The original contributions presented in the study are included in the article/Supplementary Material, further inquiries can be directed to the corresponding author.
