# Supplementary material for: Electrospun MXene/polyimide nanofiber composite separator for enhancing thermal stability and ion transport of lithium-ion batteries
Source: Front Chem. 2025 Feb 21;13:1555323. doi: 10.3389/fchem.2025.1555323 (PMC11885268; doi:10.3389/fchem.2025.1555323)
Supplement: Supplementary file 1 [file DataSheet1.docx]

**Supporting Information**

**Electrospun MXene/Polyimide Nanofiber Composite Separator for Enhancing Thermal Stability and Ion Transport of Lithium-Ion Batteries**

Yitian Wu, Wenhui Wei, Tianxue Feng, Wenwen Li, Xiaoyu Wang, Tao Wu, Xingshuang Zhang*

*Advanced Materials Institute, Qilu University of Technology (Shandong Academy of Sciences), Jinan 250014, P. R. China*

*Corresponding author. E-mail: [xszhang@qlu.edu.cn](mailto:xszhang@qlu.edu.cn) (Xingshuang Zhang)


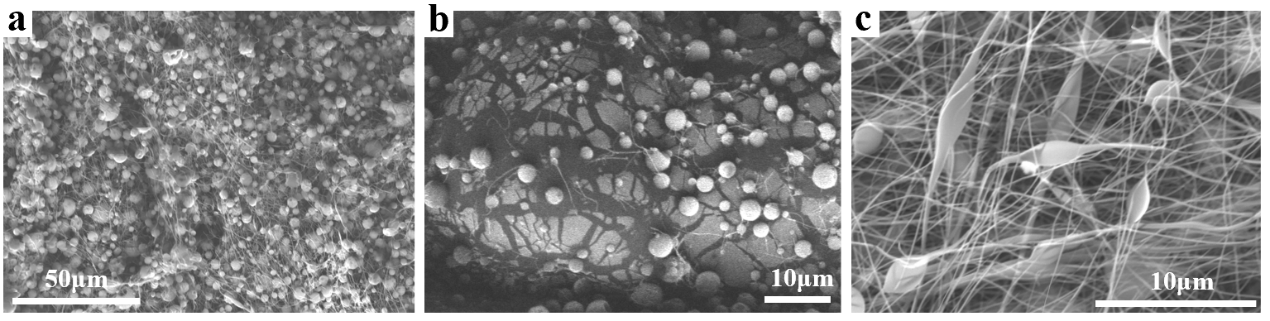


Figure S1. SEM images of PI: (a) PAA concentration is too high (40% PAA, 70% RH), (b) PAA concentration is too low (25% PAA,70% RH), (c) humidity is too high (35% PAA, 80% RH).


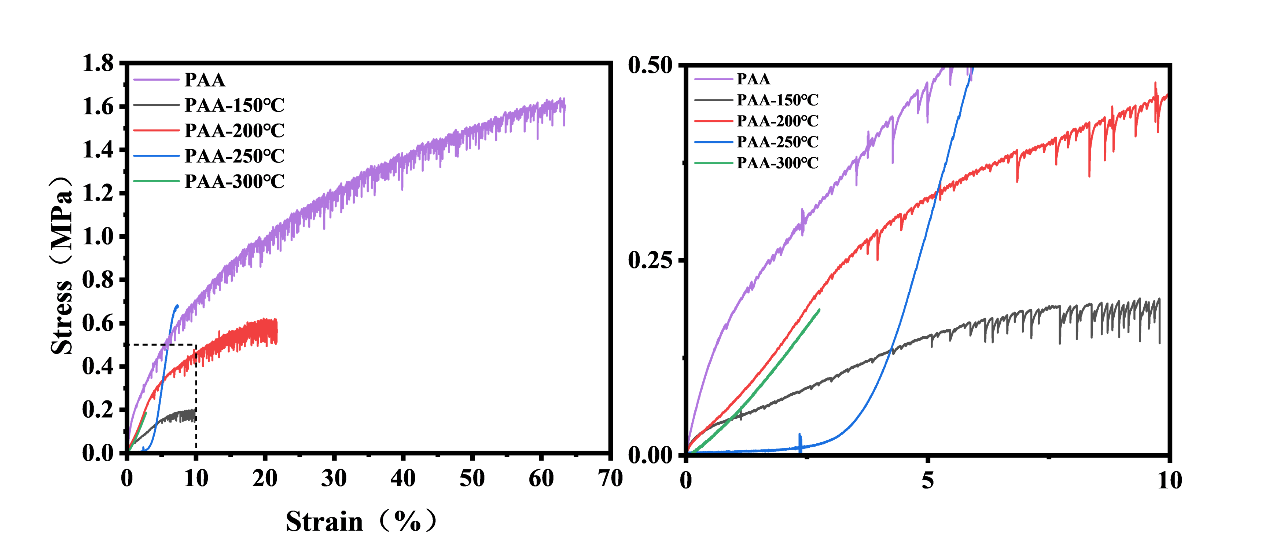


Figure S2. (a) Stress-strain curves of PI thermally imidized at different temperatures and (b) the local magnification diagram.


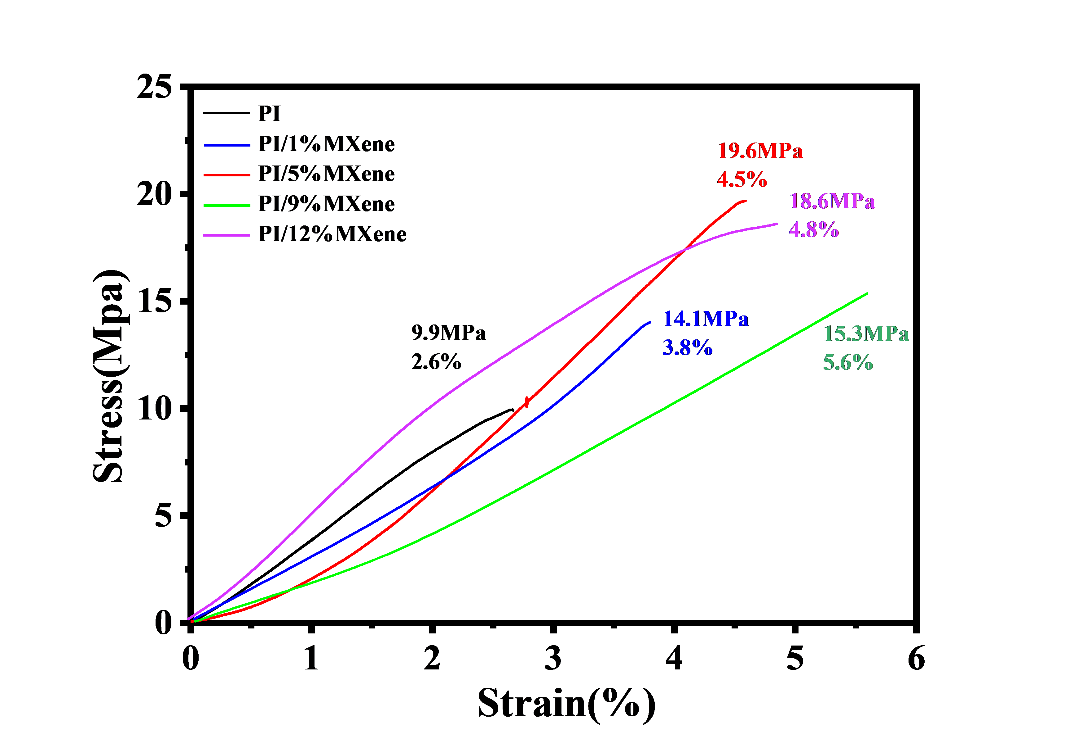


Figure S3. Stress-strain curves of PI membrane and PI/MXene membrane with different MXene doping amounts.


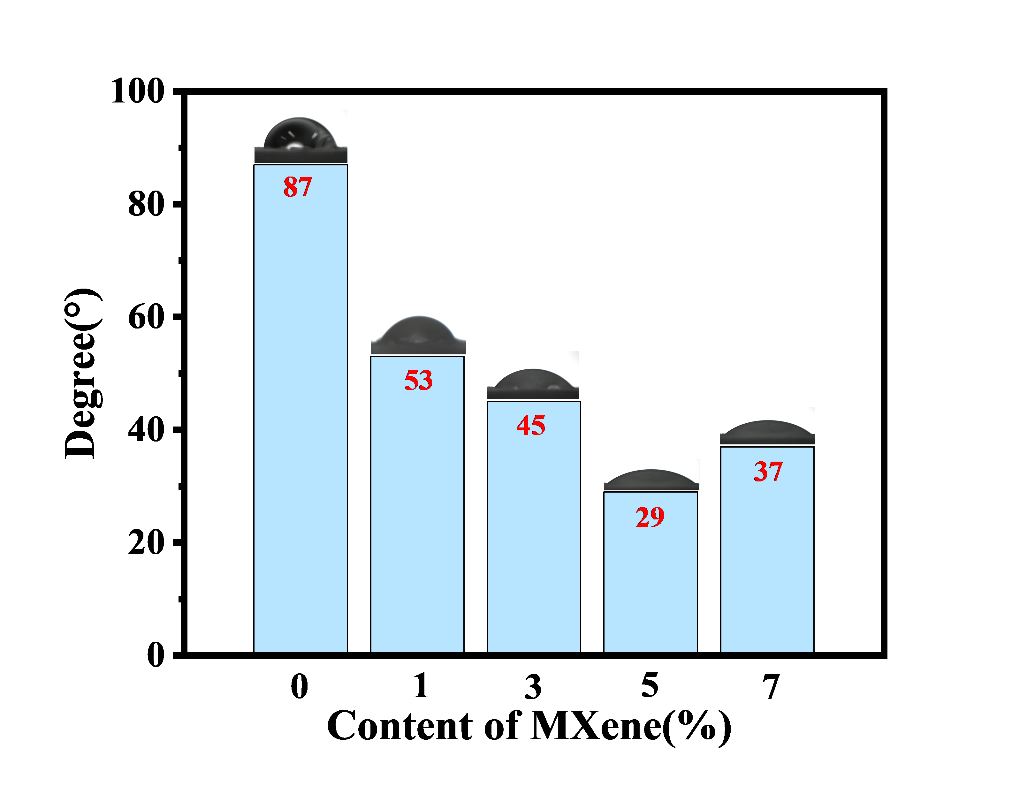


Figure S4. Electrolyte contact angles of PI/MXene membrane with different MXene doping amounts

Table S1. Combination properties of modified PI-based separators

| Materials Name | Thickness (μm) | Porosity (%) | Electrolyte uptake (%) | Tensile strength (MPa) | Discharge capacity (mAh·g^–1^) | Ref. |
| --- | --- | --- | --- | --- | --- | --- |
| Cross-linked PI | 55 | 74.3 | 279 | 37.5 | 148 (LiFePO_4_ cell, 1 C, 100 cycles) | [1] |
| SiO_2_/PAN/PI | 30 | 92 | 700 | 5.8 | 125.7 (LiFePO_4_ cell, 1 C, 100 cycles) | [2] |
| Cellulose/PI-COOH | 20 | 78 | 638 | 34.2 | 134.9 (LiFePO_4_ cell, 1 C, 100 cycles) | [3] |
| PI-COOH | 38 | 78 | 275 | 15.6 | 103.7 (LiCoO_2_ cell, 0.5 C, 100 cycles) | [4] |
| GO/PI | 40 | 68 | —— | 15.1 | 150 (LiFePO_4_ cell, 1 C, 100 cycles) | [5] |
| PI/lignin | —— | ~70 | 592 | —— | 142~135 (LiFePO_4_ cell, 0.2 C, 100 cycles) | [6] |
| **PI/MXene** | **76** | **76** | **832** | **19.6** | **126.7 (LiFePO_4_ cell, 1 C, 100 cycles)** | **This work** |

**References**

1. Kong L, Yuan L, Liu B. et al. Crosslinked Polyimide Nanofiber Membrane Prepared via Ammonia Pretreatment and Its Application as a Superior Thermally Stable Separator for Li-Ion Batteries. *Journal of The Electrochemical Society* **164**(6), 1328-1332, doi: 10.1149/2.0141707jes (2017).
2. Arifeen W, Choi J, Yook. et al. A nano-silica/polyacrylonitrile/polyimide composite separator for advanced fast charging lithium-ion batteries. *Chemical Engineering Journal* **417**, 128075, doi: https://doi.org/10.1016/j.cej.2020.128075 (2021).
3. Deng, J., Cao, D., Yang, X. & Zhang, G. Cross-linked cellulose/carboxylated polyimide nanofiber separator for lithium-ion battery application. *Chemical Engineering Journal* **433**, 133934, doi:10.1016/j.cej.2021.133934 (2022).
4. Lin, C.-E. *et al.* Carboxylated polyimide separator with excellent lithium ion transport properties for a high-power density lithium-ion battery. *Journal of Materials Chemistry A* **6**, 991-998, doi:10.1039/c7ta08702k (2018).
5. Wang, L. *et al.* Graphite oxide dopping polyimide nanofiber membrane via electrospinning for high performance lithium-ion batteries. *Composites Communications* **16**, 150-157, doi:10.1016/j.coco.2019.09.004 (2019).
6. Song, C. *et al.* A novel high-performance electrospun of polyimide/lignin nanofibers with unique electrochemical properties and its application as lithium-ion batteries separators. *International Journal of Biological Macromolecules* **246**, 125668, doi:10.1016/j.ijbiomac.2023.125668 (2023).
